# How Cooperative Engagement Programs Strengthen Sequencing Capabilities for Biosurveillance and Outbreak Response

**DOI:** 10.3389/fpubh.2021.648424

**Published:** 2021-03-01

**Authors:** Andrew W. Bartlow, Earl A. Middlebrook, Alicia T. Romero, Jeanne M. Fair

**Affiliations:** Biosecurity and Public Health, Los Alamos National Laboratory, Los Alamos, NM, United States

**Keywords:** cooperative threat reduction, emerging diseases, next generation sequencing, pandemic preparedness, One Health

## Abstract

The threat of emerging and re-emerging infectious diseases continues to be a challenge to public and global health security. Cooperative biological engagement programs act to build partnerships and collaborations between scientists and health professionals to strengthen capabilities in biosurveillance. Biosurveillance is the systematic process of detecting, reporting, and responding to especially dangerous pathogens and pathogens of pandemic potential before they become outbreaks, epidemics, and pandemics. One important tool in biosurveillance is next generation sequencing. Expensive sequencing machines, reagents, and supplies make it difficult for countries to adopt this technology. Cooperative engagement programs help by providing funding for technical assistance to strengthen sequencing capabilities. Through workshops and training, countries are able to learn sequencing and bioinformatics, and implement these tools in their biosurveillance programs. Cooperative programs have an important role in building and sustaining collaborations among institutions and countries. One of the most important pieces in fostering these collaborations is trust. Trust provides the confidence that a successful collaboration will benefit all parties involved. With sequencing, this enables the sharing of pathogen samples and sequences. Obtaining global sequencing data helps to identify unknown etiological agents, track pathogen evolution and infer transmission networks throughout the duration of a pandemic. Having sequencing technology in place for biosurveillance generates the capacity to provide real-time data to understand and respond to pandemics. We highlight the need for these programs to continue to strengthen sequencing in biosurveillance. By working together to strengthen sequencing capabilities, trust can be formed, benefitting global health in the face of biological threats.

## Introduction

Emerging and re-emerging infectious diseases are a major challenge for public health and economic security worldwide (1). Zoonotic diseases, those pathogens that can infect both animals and humans, pose the greatest risk to humans. Changes in environmental conditions (e.g., climate change and habitat degradation), biodiversity loss, habitat encroachment, and increased globalization all increase the risk that novel pathogens will spill over into humans (1–4). Preventing and dealing with the consequences of these diseases requires a global effort. Cooperative engagement programs are designed to build and strengthen capabilities and capacities in biosurveillance, biosecurity, and biosafety around the world. These programs aim to create, foster, and sustain international collaborations among health professionals, disease diagnostic laboratories, and infectious disease scientists (5, 6). They primarily focus on especially dangerous pathogens and pathogens of pandemic potential, and include those that may be used in bioterrorism. These programs have been implemented in the United States, Canada, Germany, and other countries with the goal of reducing the threat of infectious diseases by promoting global health security.

One aspect of cooperative engagement programs is to build, promote, and strengthen biosurveillance capabilities. Biosurveillance is “the ongoing systematic collection, analysis, and interpretation of health data, essential to the planning, implementation, and evaluation of public health practice, closely integrated with the timely dissemination of these data to those who need to know and linked to prevention and control” (7, 8). Early detection of pathogens is crucial for mitigation efforts and to limit the spread of a pathogen before it grows from a small outbreak to a larger epidemic or pandemic. One critical tool in biosurveillance and early detection has now become sequencing. Sequencing is not just a critical tool in biosurveillance, it can also play a major role in pandemic response. Here, we discuss the importance of cooperative engagement programs in strengthening sequencing capabilities in countries around the world.

## Cooperative Engagement Programs Strengthen Biosurveillance and Research Capabilities

**Figure 1:**
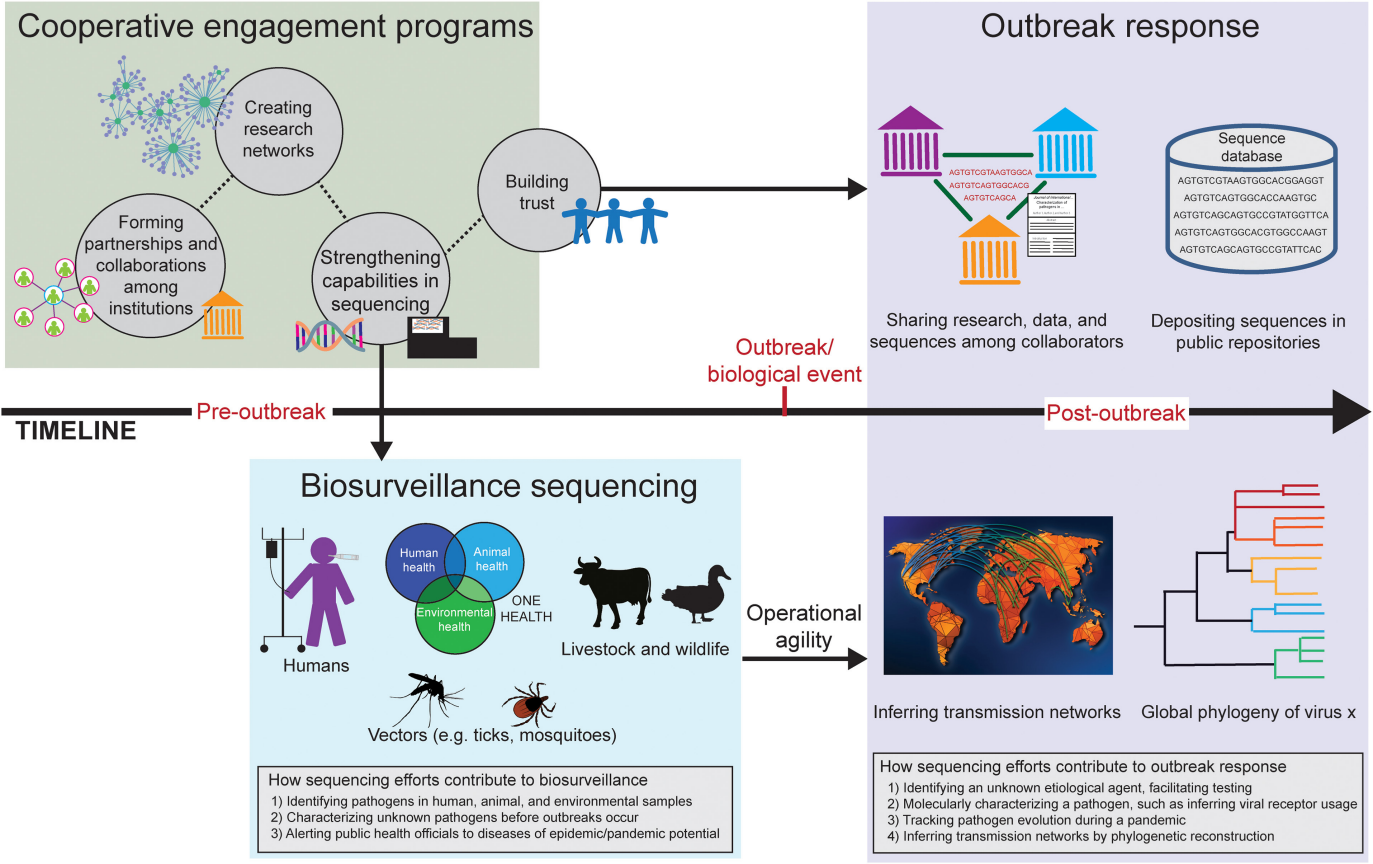
Timeline of how cooperative engagement programs strengthen sequencing efforts in biosurveillance and promote trust in outbreak response. Before an outbreak occurs (pre-outbreak), cooperative engagement programs form relationships and build collaborations among institutions in various countries (green box). Research networks can be created, offering the ability to engage in collaborative research on a particular disease system. Strengthening sequencing capabilities allows countries to use sequencing and bioinformatics in a biosurveillance capacity (blue box). Cooperative engagement programs build trust, which benefits the wider scientific community after an outbreak (post-outbreak; purple box), such as sharing research and pathogen sequences. Having sequencing technology in place for biosurveillance allows for operational agility in the event of an outbreak; knowledge and resources can be co-opted for epidemic and pandemic response, such as providing data to infer transmission networks and create global phylogenies.

Cooperative engagement programs bring together researchers, scientists, and health professionals from all over the world with the shared goal of reducing the threat of infectious diseases (5, 6). These programs help to establish and strengthen both biosurveillance efforts and research capabilities to answer important questions regarding infectious diseases (Figure 1). A vital consequence of these programs is trust. Trust is formed in the initial capacity building for sequencing and biosurveillance through reachback efforts. Reachback is the technical training and support from partner institutions. Participating countries and institutions can also help each other to perform sequencing and to analyze sequence data, which further enhances relationships. Once the sequencing capability is built, sustaining this capability is challenging; sequencing technology and bioinformatics tools are constantly changing and labs need to keep purchasing supplies and reagents. Collaborations, research networks and projects, and reachback support are important pieces to sustainability. These allow participating countries to continue to actively use sequencing and bioinformatics tools to answer important questions regarding, for example, pathogen biology and disease distribution. Cooperative engagement programs help build the initial capacity and continue to play a role in sustaining these capabilities.

Every country is at a different level of readiness for sequencing and genomics, from laboratory expertise to computational and bioinformatics proficiency. Various funding agencies assess if sequencing is appropriate and is able to be sustained in a given country. Difficulties in building a genomics capability include cost of equipment, obtaining service contracts for instruments, obtaining kits and reagents in country, sustaining a trained workforce, and having the proper infrastructure. For low-income institutions, funds from sponsors help with the initial capacity building and then through collaborative research projects. Once partnerships are formed, cooperative engagement programs foster trust by encouraging participants to work together side-by-side to solve problems and challenges. Other trust-building activities include trainings, mentoring and advising in professional development, and developing personal relationships through in-person meetings. All of these activities increase the chances that a genomics capacity will be developed and used in biosurveillance.

Collaborations among researchers and institutions can lead to the creation of research networks (Figure 1). These research networks are typically centered around a common pathogen, disease, or host of interest, such as a research network on bat-borne viruses or Rickettsial pathogens (9). Often called “threat reduction research networks,” they increase sustainability and maintain relationships. Trust is key to creating and sustaining networks, which allows for data sharing among collaborators within the network. Threat reduction research networks encourage collaborations on specific research projects, thereby creating new connections and maintaining those already established. The expertise of each institution can be leveraged; and therefore, they can provide support to each other throughout the duration of a funded research project. With increased travel and globalization, the world is more connected than ever, highlighting the importance of the saying “a threat anywhere is a threat everywhere” (6). Having threat reduction research networks in place with the trust that has been formed benefits the broader scientific community, such as in the early stages of an epidemic or pandemic through data sharing (Figure 1).

Cooperative engagement programs support and promote the One Health initiative (Figure 1). One Health is the integrative and multidisciplinary approach to reducing disease risk by focusing on the health of people, animals, and the environment. A One Health approach to biosurveillance and threat reduction research networks is an important framework for understanding the whole ecology of disease systems, from wildlife reservoir hosts to the role of environmental conditions and habitat disturbances (10). We need to be able to reliably identify pathogens of importance in humans and domestic animals. Wildlife and changes in biodiversity need to be understood and addressed as well, which is an often-overlooked component of the global health security agenda (11, 12). The whole ecology of a disease system leads to a better understanding of the disease and provides information to enhance the effectiveness of biosurveillance efforts. This knowledge maximizes limited resources by allowing researchers to target specific hosts and environments important to the pathogen in question (13).

## Sequencing in Biosurveillance

Sequencing is an essential and critical tool in biosurveillance (14). In biosurveillance, early detection is crucial. Identifying pathogens in human, animal, and environmental samples cannot be completed without next generation sequencing. Sequencing is being introduced to and adopted by countries all over the world. Democratization of sequencing is a crucial aspect of threat reduction by making sure all countries can afford to adopt this technology and by making the process of sequencing and the analysis of genomic data more feasible (15). Next generation sequencing is still challenging to adopt because of the high learning curve associated with understanding the theory and techniques and gaining practical experience.

In an effort to build sequencing capabilities in partner countries, trainings and workshops led by partner institutions are needed to teach both “wetlab” techniques and bioinformatics. Wetlab topics include sample collection, storage, and preparation; DNA and/or RNA extraction; and performing a sequencing run. Along with trainings in these wetlab techniques, cooperative engagement programs promote training in applying bioinformatics tools to genomic data. Bioinformatics tools are essential to analyzing sequence data. Quality control, taxonomy identification tools (e.g., Kraken2, GOTTCHA2, MetaPhlAn2), phylogenetics, and reference-based analysis are all needed to identify and characterize pathogens in a particular sample and to get the most information out of genomic data as possible. Software such as Los Alamos National Laboratory's EDGE (Empowering the Development of Genomics Expertise) Bioinformatics platform (www.edgebioinformatics.org) are designed to make the analysis of genomic data relatively straightforward (16).

An essential component of sequencing is the collection and inclusion of metadata. Metadata are the information that describe a given sample. Metadata are important throughout the entire life of a sample; from sample collection through sequencing and bioinformatic analyses. When a sample is first collected, important metadata include, for example, time of collection, what kind of sample it is (e.g., throat swab, fecal sample, blood sample), where the sample was taken, from what host species it came, and location and environmental data (e.g., GPS coordinates and habitat type). Extraction methods, library preparation, and sequencing platform are also important to include as the sample is processed. Furthermore, recording bioinformatic analyses used to process data helps researchers use data that are deposited in public repositories. Integration of metadata with genomic data is key when adding it to a repository, especially linking raw data and processed data that may end up in different databases. These data are extremely important for research related to biosurveillance and sequencing pathogens during a pandemic, and help with contextualizing sequencing data and decision making. For example, without the proper metadata, conclusions regarding pathogen origin and evolution would not be possible. Metadata are especially vital for samples that are in long-term storage that may be processed at a later date.

Applying sequencing and bioinformatics to biosurveillance and threat reduction research networks is key to understanding the whole ecology of disease systems in a One Health context. Knowledge of potential reservoir hosts and/or vectors, the circumstances of transmission events, and the effects of environmental conditions helps to reduce the risk of infectious disease threats and provides information to enhance biosurveillance efforts and effectiveness (17). For example, shotgun metagenomics and metatranscriptomics, and associated taxonomic ID tools, can be used to identify known and novel pathogens in human, animal, and environmental samples. By surveying the types of pathogens present in different regions, public health officials can be alerted about a potential threat and limit the severity and cost of an epidemic or pandemic (18) (Figure 1).

Putting the sequencing systems in place and conducting trainings before an epidemic or pandemic occurs gives operational agility to respond to an outbreak and help with mitigation efforts (Figure 1). The technology and training need to be put in place prior to public health crises. Once in place, the technology can be co-opted to pandemic response; only the goals and questions being addressed change. Cooperative engagement programs begin establishing relationships and developing collaborations before an outbreak or a biological event occurs. Setting up sequencing machines and training local staff members how to go from sample to sequence to analysis is the first step. By investing time and effort into strengthening sequencing and bioinformatics capabilities through partnerships, trust is built, which can be leveraged both for biosurveillance (pre-outbreak) and outbreak response (post-outbreak; Figure 1).

## Benefits of Sequencing Throughout a Pandemic

A major tool in the fight against a pandemic is pathogen sequencing. Whereas, sequencing in a biosurveillance capacity deals with questions related to identifying and characterizing pathogens before outbreaks occur, sequencing throughout a pandemic provides real-time data on pathogen transmission and evolution (Figure 1). The trust gained from and promoted by cooperative engagement programs plays a major role in the sharing of sequence data among institutions and countries, as well as depositing sequences in public repositories (e.g., GISAID [https://www.gisaid.org]) (Figure 1). Having the technology and tools allows for operational agility; if the tools for biosurveillance are in place, it allows institutions to shift to pandemic response (Figure 1). In general, global sequencing efforts during an epidemic or pandemic can (1) identify unknown etiological agents, facilitating testing; (2) molecularly characterize pathogens such as inferring viral receptor usage; (3) track pathogen evolution throughout the duration of a pandemic; and 4) infer transmission networks by phylogenetic reconstruction. This information can be used to guide effective intervention strategies and track response efforts over time.

Early in pathogen emergence, quickly identifying the etiological agent is critical for developing diagnostic tools, slowing transmission, guiding patient care, and designing therapeutics. For some agents, microbiological techniques such as microscopy and differential growth media can identify pathogens. These techniques likely lead to a broad understanding of the pathogen identity (i.e., bacterial genus, virus family). If genetic data are available for the pathogen group, PCR can be used to identify/confirm the pathogen identity. Using next-generation shotgun sequencing is a much faster and specific way to identify a pathogen requiring little *a priori* knowledge. Thus, sequencing patient samples with instruments like Oxford Nanopore's MinION or Illumina's MiSeq can identify pathogens, including those that are well-known, emerging, or completely novel, days earlier than conventional microbiological and sanger sequencing methods. For COVID-19, the pathogen genome was sequenced and compared to available sequences on public databases (19, 20). This revealed it was related to previously sequenced human pathogenic coronaviruses, such as the viruses that caused the 2003 SARS and 2012 MERS epidemics (21). However, it showed greater similarity to a coronavirus sequence obtained from bats (19). Interestingly, the spike protein, responsible for viral entry into cells, was more closely related to a separate published coronavirus sequence identified in pangolins (22). This paved the way for a more nuanced understanding of the evolutionary origins of the novel coronavirus responsible for the pandemic. Having a large amount of cataloged sequence data allowed for these conclusions to be made quickly, informing decision-making regarding mitigation efforts.

The number of microorganisms in sequence databases is staggering, and the more broadly researchers sample, the more prepared the scientific community will be to respond to novel emerging pathogens. Collaborative engagement programs and threat reduction networks have the ability to generate sequencing data from understudied regions, closing a knowledge gap in sequence databases. Leveraging molecular studies of related pathogens to understand a current epidemic or pandemic pathogen is predicated on knowing its genome sequence. The genome sequence of SARS-CoV-2 allowed the inference of host receptor usage. Based on similarity to related coronaviruses, especially SARS-CoV-1, it was likely the virus used the angiotensin-converting enzyme 2 (ACE2) of human lung cells to gain entry (23, 24). This initial understanding, plus other homology-based insights, led to a list possible therapeutics (25), with current therapeutic options and promising candidates [reviewed in (26)].

With each additional country that participates in sequencing a pandemic pathogen, the power to identify loci contributing to adaptation to humans increases. Every transmission cluster that has sequences available is an independent evolution experiment. Variants emerging during a pandemic are of great concern because they can affect viral virulence, immunogenicity, and transmission, but also can impact diagnostic tests and vaccine effectiveness. For instance, 10,000 sequences deposited in GISAID (27) were used to assess that SARS-CoV-2 appears not to be responding to human T-cell immune pressure, but is responding to B-cell epitopes, indicating humoral immunity imparts a significant selective pressure on the virus (28). Genome sequences from all over the world being deposited in GISAID allowed Korber et al. (29) to show that the emergent variant leading to the amino acid change D614G of the coronavirus spike protein, which came to dominate SARS-CoV-2 sequences, is more transmissible. They went on to link clinical data to viral genome sequences and show that the increased transmissibility is likely due to increased viral shedding. Having many publicly available pathogen sequences can also help researchers predict the robustness of diagnostic tools to pathogen evolution (30). All of these research directions are critical for a real-time, robust response to an ongoing pandemic, and are greatly strengthened by sequence contributions across all geographic regions, including those engaged in cooperative biological engagement programs.

During a pandemic, one of the most vital contributions of collaborative sequencing efforts is to expand the number and geographic distribution of sequenced samples. Sequencing labs originally set up to address local outbreaks of endemic pathogens, can rapidly pivot to sequencing samples of a pandemic pathogen. For many countries, cross border movement of goods and workers is essential to the economy, individual livelihoods, and more importantly food distribution (31). Therefore, evidence-based decision making is critical. Identifying sources of new infections across country borders can be very difficult with traditional tracking and tracing methods, especially where infrastructure is limited. Thus, it is hard to know the efficacy of shutting down international travel for preventing pathogen spread. Phylogenetic analysis of sequences from an ongoing pandemic can identify if cases are from local transmission events or imported from other countries through human travel (32–34). These analyses are predicated on robust sequencing operations across regions including all countries with travel ties; the more sequencing across a region, the more everyone benefits. This makes collaborative efforts to stand up sequencing infrastructure an important component in decision making during an ongoing pandemic.

## Discussion

Emerging and re-emerging diseases will continue to spread to new areas and affect millions of people around the world. Even without including viruses with pandemic potential (e.g., coronaviruses and influenza viruses), zoonotic infectious diseases affect tens of millions of people per year and cause substantial economic and human health impacts (1, 4, 18, 35). These diseases often emerge with no warning, and as a result, health officials in charge of mitigation efforts begin at a disadvantage. In response to the threats of unforeseen pathogens, effective biosurveillance needs to be implemented. The goal of cooperative engagement programs is to increase the ability and capacity to detect and respond to infectious disease outbreaks, specifically those involving especially dangerous pathogens and pathogens of pandemic potential.

Cooperative engagement programs help to strengthen both biosurveillance and research capabilities to answer important questions regarding infectious diseases. An important component of these programs is building trust among institutions and countries by strengthening sequencing capabilities together. Sustaining the collaborations and capabilities through threat reduction research networks and collaborative research projects further builds trust among researchers, institutions, and countries. This enables sample and data sharing, which is critical throughout the duration of an epidemic or pandemic. We urge the continued effort of cooperative engagement programs to strengthen sequencing capabilities for biosurveillance and research regarding emerging and re-emerging diseases. We need global unity in the fight against emerging diseases that pose significant risk to global health security, and cooperative engagement programs are leading the way.

## Data Availability Statement

The original contributions presented in the study are included in the article/supplementary material, further inquiries can be directed to the corresponding author/s.

## Author Contributions

JF and AB conceived the idea of the manuscript. AB and EM wrote the first draft of the manuscript. JF and AR provided critical comments and edits. All authors contributed to manuscript revision, read, and approved the submitted version.

## Conflict of Interest

The authors declare that the research was conducted in the absence of any commercial or financial relationships that could be construed as a potential conflict of interest.
